# Consultation on UTUC, Stockholm 2018 aspects of risk stratification: long-term results and follow-up

**DOI:** 10.1007/s00345-019-02739-1

**Published:** 2019-04-03

**Authors:** Mudhar N. Hasan, Morgan Rouprêt, Francis Keeley, Cecilia Cracco, Robert Jones, Michael Straub, Olivier Traxer, Palle Jörn Sloth Osther, Marianne Brehmer

**Affiliations:** 1 Division of Urology, Department of Clinical Sciences, Danderyd Hospital, Karolinska Institutet, Stockholm, Sweden; 2 Sorbonne Université, GRC n°5, ONCOTYPE-URO, AP-HP, Hôpital Pitié-Salpêtrière, 75013 Paris, France; 3 Bristol Urological Institute, Southmead Hospital, Bristol, England UK; 4 Department of Urology, Cottolengo Hospital of Torino, Torino, Italy; 5 Institute of Cancer Sciences, University of Glasgow, Beatson West of Scotland Cancer Centre, Glasgow, UK; 6 Department of Urology, University Hospital Klinikum Rechts Der Isar, Technical University Munich, Munich, Germany; 7 Sorbonne Université, Hôpital Tenon, Paris, France; 8 Department of Urology, Lillebaelt Hospital, University of Southern Denmark, Vejle, Denmark

**Keywords:** Urothelial carcinoma, Upper tract, UTUC, Treatment, Risk stratification, Follow-up, Prognosis, KSS, RNU, Renal pelvis, Ureter

## Abstract

**Purpose:**

To summarize current knowledge on upper urinary tract carcinoma (UTUC) regarding risk stratification, long-term results, and follow-up.

**Methods:**

A scoping review approach was applied to search literature in Pubmed, Web of Science, and Embase. Consensus was reached through discussions at Consultation on UTUC, September 2018, Stockholm.

**Results:**

To optimize oncological outcome considering both cancer-specific survival (CSS) and overall survival (OS), it is essential to identify pre- and postoperative prognostic factors. In low-risk UTUC, kidney-sparing surgery (KSS) and radical nephroureterectomy (RNU) offer equivalent CSS, whereas RNU may result in poorer OS due to nephron loss. For more aggressive tumours, undergrading can lead to insufficient treatment. The strongest prognostic factors are tumour stage and grade. Determining grade is best achieved by ureterorenoscopy (URS) with focal samples, biopsy and cytology. Staging is more difficult but can be indirectly achieved by multiphase computed tomography urography (CTU) or tumour grade determined by cytology and histopathology. Patients treated with KSS should be monitored closely with regular follow-ups (URS and CTU).

**Conclusion:**

KSS should be offered in low-risk UTUC when feasible, whereas RNU is the treatment of choice in organ-confined high-risk UTUC. Intravesical recurrence (IVR) is common after RNU, but a single postoperative dose of mitomycin instillation decreases IVR. Endourological management has high local and bladder recurrence rates; however, its effect on CSS or overall survival OS is unclear. RNU is associated with significant risk of chronic kidney disease. Careful selection of patients and risk stratification are mandatory, and patients should be followed according to strict protocols.

## Introduction

Although urothelial carcinoma is the fourth most common tumour type, urothelial carcinoma of the upper urinary tract (UTUC) is rare and accounts for only 5–10% of urothelial cancers [1], with an estimated annual incidence of nearly two cases per 100,000 inhabitants in Western countries. Smoking is the most important risk factor for UTUC, increasing the relative risk (RR) 2.5- to 7-fold [2]. Unlike bladder urothelial carcinoma where approximately 25% are invasive at diagnosis, 40–60% of UTUCs are invasive at diagnosis [3, 4]. UTUC is more common at the ages greater than 70 years, and is three times more common in men.

The current guidelines outlined by the European Association of Urology (EAU) divide UTUC into low- and high-risk disease [5]. The goal of risk stratification is to reduce the risks of understaging, undergrading, and overtreatment, and to select the best treatment option for long-term health.

Radical nephroureterectomy has previously been the gold standard for treatment of UTUC. However, with the emergence of endoscopic techniques, a number of studies have indicated that kidney-sparing surgery (KSS) and treatment with radical nephroureterectomy (RNU) offer equivalent long-term CSS in patients with low-risk UTUC [6, 7]. RNU is recommended for organ-confined high-risk UTUC [5]. Inasmuch as UTUC is a relatively rare disease, most recommendations for clinical decision-making are based on retrospective studies. Tumour stage and grade have proven to be the strongest prognostic factors [3, 8], and some studies have reported that size and multifocality are not as decisive [9].

**Fig. 1:**
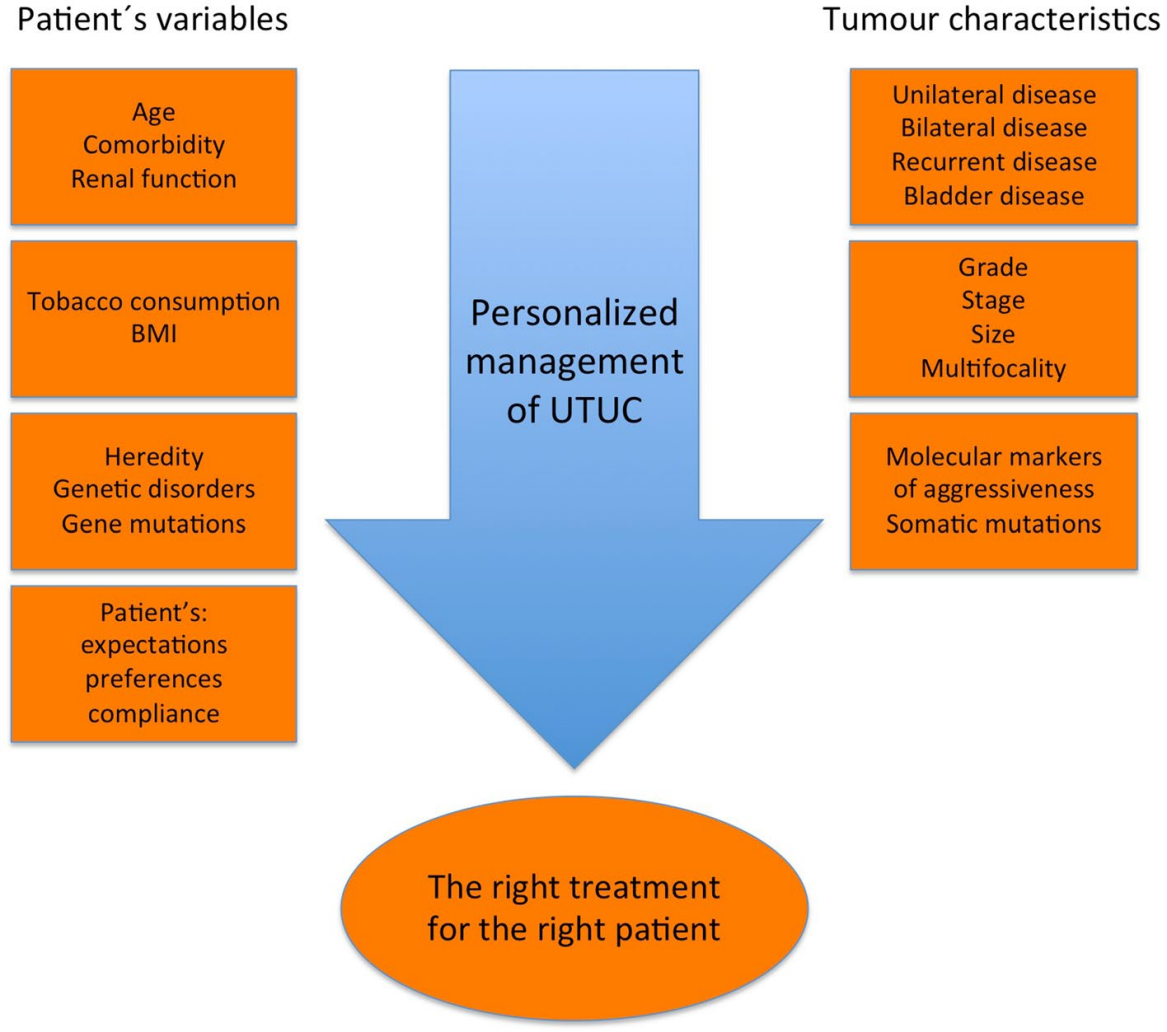
Personalized management of UTUC. Before making a treatment decision, all variables must be taken into consideration, including patient-related aspects and tumour biology. *BMI* body mass index

When considering treatment options, it is essential to correctly identify high-risk UTUC, because KSS can represent undertreatment, and RNU unequivocally results in loss of renal function and risk of chronic kidney disease (CKD), both of which are known to be associated with increased risk of morbidity and mortality [10]. Thus, both prognostic tumour characteristics and general health variables of the patient should be taken into consideration (Fig. 1). The choice of treatment also depends on whether an approach is regarded as elective, relative, imperative, or palliative.

Clearly, risk stratification must be optimized to achieve safe and personalized management of UTUC.

This review aims to consider the challenges in risk stratification of UTUC by discussing different prognostic factors, follow-up, and long-term results after different treatment modalities in UTUC by expanding the current recommendations, and showing directions for future research. A scoping review approach was applied to search literature in Pubmed, Web of Science, and Embase.

### Risk stratification

To predict prognosis and determine whether KSS might be more beneficial than radical extirpative surgery, risk stratification is a very important part of the clinical decision-making for patients with UTUC [11].

As mentioned above, the EAU guidelines categorize UTUC into low- and high-risk diseases [5]. Low-risk UTUC is defined as follows (all criteria must be fulfilled): a unifocal tumour with a size of < 2 cm, low-grade cytology and histology (URS biopsy), and non-invasive aspects on computed tomography urography (CTU). High-risk UTUC is defined as the presence of one or more of the following criteria: hydronephrosis, tumour size > 2 cm, high-grade cytology or histopathology (biopsy), multifocal disease, or previous radical cystectomy for bladder cancer.

Other preoperative risk factors that should be taken into consideration are tobacco consumption, advanced age, BMI > 30, and presence of comorbidity.

Nevertheless, both preoperatively and postoperatively, by far the strongest prognostic factors are stage and grade, followed by presence of carcinoma in situ (CIS), performance of bladder cuff excision or not at RNU (where not performing bladder cuff excision increases the risk), lymphovascular invasion, lymph node involvement, tumour architecture, positive surgical margins, tumour necrosis, molecular markers, and histological variant [5].

The best tool for preoperative grading is ureterorenoscopy with simultaneous collection of samples for cytology and histopathology [12–14]. Also, various models have been proposed for further risk stratification. Favaretto et al. [15] published a scoring system for the prediction of muscle-invasive and non-organ-confined UTUC, which consisted of a preoperative multivariable model that combined ureteroscopy variables and imaging. This system has not been validated by the researchers. Rouprêt and co-workers [16] developed a nomogram for the prediction of CSS after RNU for UTUC; postoperative variables are plotted in the nomogram, and the 5-year CSS is calculated. A limitation of this system is that it uses postoperative prognostic calculations that cannot aid preoperative treatment decisions.

As mentioned, UTUC is a rare disease, and hence scientists have often tried to draw comparisons between UTUC and bladder cancer. However, in reality, the two diseases are like disparate twins, that is, they share many features but differ in several aspects, including anatomical, biological, and molecular dissimilarities that warrant consideration in clinical decision-making [17]. Another risk factor for UTUC is hereditary non-polyposis colon cancer (HNPCC) or Lynch syndrome, which should be suspected in patients aged < 60 years or with a personal history of HNPCC-spectrum cancer. Patients with HNPCC have a cumulated risk of 1–28% of developing UTUC during their lifetime [18]. Nearly 20 years ago, tumour microsatellite instability (MSI) was first described as a predictor of survival in 95% of all patients with HNPCC syndrome [19]. In 2005, Rouprêt et al. [20] studied the impact of MSI in advanced UTUC and found that the survival rate was higher in patients who had MSI than in those who did not. The role of germline DNA mutations has also been discussed. It has been shown that genetic polymorphisms on 8q24.1 and 4p16.3 are associated with aggressive UTUC but not with bladder cancer. It has also been reported that genetic variability in 8q24 leads to greater risk of developing UTUC, and that the T/T genotype is associated with aggressiveness of this disease. Moreover, a retrospective study of 83 tumours revealed that only two alterations were uniformly associated with high grade and advanced stage of a tumour [21]: TP53/MDM2 alterations, which were associated with poor prognosis, and FGFR3 mutations, which were associated with more favourable outcome.

### Long-term results

Most surgical series have limited follow-up and are retrospective, and thus it can be difficult to compare the results. The EAU guidelines panel [22] reviewed 42 studies that compared open and laparoscopic RNU. A total of 7554 patients were included in the 42 investigations (median 36 patients per arm), and the techniques used varied. The risk of bias and confounding was high in most of the reviewed studies. Only one of the 42 investigations was a randomized clinical trial (*n* = 80) [23], and only four of the studies (including a total of 300 patients) had at least 5 years of follow-up [24–27]. Furthermore, only nine of the studies adjusted for risk factors. Port-site metastasis rates ranged from 0% to 2.8%. The EAU guidelines panel concluded that open RNU may be a better choice for patients with pT3/pT4 disease.

Seisen et al. [28] conducted a multi-centre retrospective analysis of 304 patients who had unifocal distal ureteral tumours treated with RNU (*n* = 128), distal ureterectomy (*n* = 134), or endoscopic ablation (*n* = 42). The mean follow-up time was 30.7 months. The results showed that OS was better for patients treated with KSS (i.e., ureterectomy or ablation), although CSS was similar for all three treatment groups. In another evaluation, the EAU guidelines panel [29] reviewed 22 retrospective studies that compared RNU with different methods of KSS [i.e., segmental ureterectomy, ureterorenoscopy (URS), or a percutaneous approach] for treatment of UTUC. This analysis revealed that survival in patients with low-grade and non-invasive UTUC after RNU was similar to survival noted after KSS with URS or the percutaneous approach. Furthermore, considering high-grade and invasive UTUC, selected patients benefited from selected ureterectomy (when feasible), with similar oncologic outcomes as after RNU. Nevertheless, it can be concluded that, in general, the data presented in the reviewed studies were poorly reported, and there was a selection bias in favour of KSS.

Grasso et al. [6] summarized 15 years of experience of 160 UTUC patients treated between January 1996 and August 2011. The patients were divided into three distinct groups based on endoscopic samples and treatment type: group 1, low-grade lesions treated with URS (*n* = 66); group 2, high-grade lesions palliatively treated with URS (*n* = 16); group 3, high- or low-grade lesions treated with extirpative surgery (RNU; *n* = 80). The patients with low-grade disease treated with URS with curative intent (group 1) had 2-, 5-, and 10-year CSS rates of 98%, 87% and 81%, respectively; the corresponding figures for patients treated with RNU (group 3) were 97%, 87%, and 78%. The patients with high-grade disease and treated with RNU had 2-, 5-, and 10-year CSS of 70%, 53%, and 38%, and metastasis-free survival of 55%, 45%, and 35%, respectively. For patients with high-grade disease treated with palliative URS, median survival was 29.2 months, and 2-year OS was 54%. Ten patients who initially had low-grade UTUC treated with KSS progressed to high-grade disease at a mean time of 38.5 months.

In another paper, Cutress et al. [30] described 20 years of experience of endoscopic treatment of UTUC in 73 patients with a mean follow-up of 62.8 months. Disease-specific survival was 88.9% at 5 years and 77.4% at 10 years for all grades. Furthermore, outcome was much better for grade 1 (G1) tumours than for grade 3 (G3) tumours with regard to disease-specific survival, upper tract and bladder recurrence-free survival, renal unit survival, upper tract progression-free survival, and endoscopic failure-free survival. The results for grade 2 (G2) tumours were in between those noted for G1 and G3 lesions.

Keeley et al. [31] published the outcomes in 38 patients (41 kidneys) treated with KSS and followed endoscopically for a mean of 35 months. Tumour grading was possible in 40/41 kidneys. Low-grade tumours (G1 or G1–G2) were found in 21 renal units (19 patients), G2 in 14, and G3 in five. The patients had undergone more than 200 procedures and more than 90 treatments with no local progression, no metastatic disease, and no cancer mortality.

Petros et al. [32] reviewed the oncological outcome of retrograde endoscopic management of UTUC in 15 published reports. The inclusion criterion was a minimum of 2 years of follow-up. The studies differed with regard to the number of patients included (10–82, mean 40, median 35), and the total number of patients was 597. Recurrences were observed in the upper tract in 65% of the patients and in the bladder in 44%. OS was 35–100%, whereas CSS was 70–100%, possibly due to disparities in the spectrum of tumour grades treated in the various studies. In six of the investigations, CSS was 100%. Four of those six studies included only low-grade tumours in 35, 25, 10, and 10 patients, respectively [33–36]. The study with the lowest CSS (70%) had a large number of high-grade tumours (two G1, 13 G2, and seven G3), whereas the other studies mainly included low-grade tumours. Few complications were reported, primarily strictures. Petros et al. concluded that all data supporting endoscopic management were based on level 3 evidence, with no prospective studies available. The endoscopic ablative technique was clearly presented in only 8 of the 15 studies, was briefly mentioned (with references) in three of the studies, and was not mentioned at all in four. However, in the investigations that did consider this technique, holmium (Ho):YAG and Ho:YAG in combination with Neodymium:YAG was applied most often.

Painter et al. [37] have reported how they changed their selection criteria for KSS in patients with UTUC over the years. These investigators compared inclusion criteria and the outcome of 35 ureteroscopic treatments performed during 1998–2006 with 30 procedures carried out during 2006–2016. In the earlier group, indications were broader, and there were six deaths from cancer progression. During the period 2006–2016, only endoscopic treatment of low-risk UTUC was performed, and no patients developed metastatic disease. The recurrence rate was high in both groups.

In a prospective consecutive study conducted by Villa et al. [9], 92 UTUC patients ureteroscopically treated with Ho:YAG laser were enrolled over a period of 13 years. The median follow-up time was 52 months. In this analysis, grade was an independent predictive factor of disease progression, whereas tumour size ≤ 1 or > 1 cm and multifocality were not. At 2-year follow-up, progression-free survival was 79% for low-grade and 52% for high-grade disease, and this difference was statistically significant.

Intravesical recurrence (IVR) has been reported in 27–40% of UTUC patients after RNU [38]. However, Obrien et al. [39] found that the incidence decreased significantly when a single dose of intravesical mitomycin was given postoperatively. IVR after diagnostic URS has also been suggested by several investigators, although these findings have not been found to have an impact on OS, CSS, recurrence-free survival, or metastasis-free survival [40–42]. Another study showed no correlation between previous ureteroscopic tumour ablation and IVR or cancer-specific mortality in patients treated with an upper urinary tract ablation prior to RNU [43].

### Follow-up

**Fig. 2:**
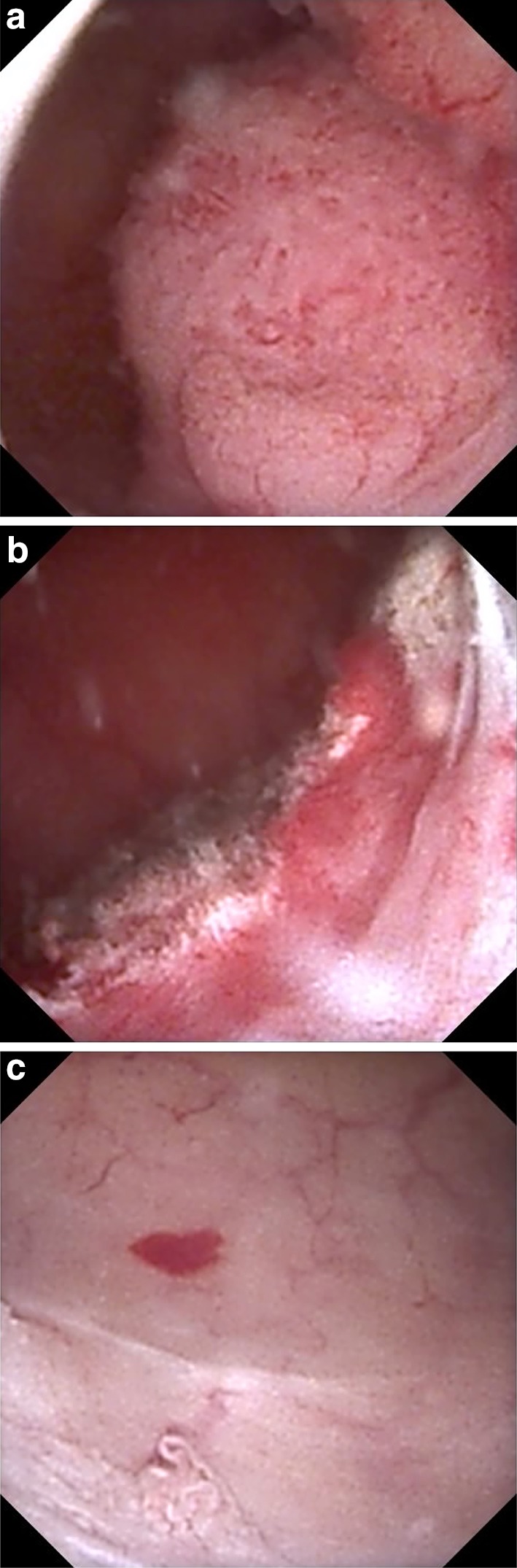
A urothelial carcinoma in the renal pelvis of an 83-y.-o man. The tumour has a surface diameter of 17 mm and there are no contraindications for RNU. The patient requested KSS, and, after being informed of the advantages and disadvantages of such treatment, that it might have to be performed in two sessions, and that it will require repeated URS, the patient accepted and underwent ureterorenoscopic Ho:YAG laser ablation. Second look at 6 weeks after the procedure showed a small residual tumour, which was laser treated. Histopathology on biopsy showed low-grade (G1–2) UTUC, and cytology also showed low-grade disease. The patient had no recurrence at follow-up 15 months after the last ablation. **a** Before ablation. **b** After first ablation. **c** At 6-week second look: one < 1 mm residual tumour was found and ablated

According to the EAU guidelines, follow-up after RNU for low-risk UTUC should include cystoscopy at 3 and 9 months and thereafter annually, as well as CTU annually. For patients with high-risk UTUC, cystoscopy with cytology should be performed every 3 months for 2 years, then every 6 months for 2 years, and thereafter annually for 5 years together with CTU annually. After KSS for low-risk UTUC, cystoscopy and CTU should be done at 3 and 6 months and then annually for 5 years, including URS at 3 months. The same programme should be applied for high-risk tumours, but adding ureteroscopy with in situ cytology at 3 and 6 months. Evidence supporting use of the mentioned follow-up regimes is weak. Other authors have suggested using both a closer and a longer follow-up. Verges et al. [44] described a follow-up protocol with URS at 3-month intervals until the patient is disease free, followed by URS at 6-month intervals for 5 years and thereafter annually, and also annual CTU. Some authors strongly recommend a second look in all cases at 6 to 8 weeks after laser ablation [45] (as illustrated in Fig. 2).

## Discussion

Radical RNU was the gold standard for treatment of all patients with UTUC until the EAU guidelines were updated during 2011–2013. Before that time, KSS was to be considered only in imperative cases. However, a better understanding of the disease and improved technology have led to increased use of kidney-sparing management of UTUC, and, along with that, there is a growing need for guidance of treatment decisions.

The EAU guidelines have divided UTUC into low- and high-risk diseases since 2015 [46]. The update presented that year stated that KSS could be considered in low-risk patients, whereas the 2018 update recommended that KSS be performed in all low-risk cases [5]. Moreover, the definition of low- and high-risk UTUC has been changed to some degree in the 2018 update. Also, the cut-off for tumour size, one of the listed prognostic factors, was initially set at < 1 cm for low-grade tumours but has now been changed to < 2 cm.

UTUC is a relatively rare disease, and most studies in the literature have been retrospective and included limited numbers of patients. Consequently, the available evidence in this context is relatively weak. Grade and stage have been shown to be the strongest prognostic factors [3, 5, 8], whereas the significance of tumour size and multifocality has been questioned [9, 47]. Direct staging is not possible, because endoscopic samples are very small, and deep biopsies are associated with the risk of perforation and tumour seeding [48]. Still, there is a strong correlation between stage and grade, which to some extent does enable indirect staging [3, 49, 50]. Clearly, it is essential to achieve correct grading. However, that task has proven to be challenging for endoscopic samples [51, 52], possibly due to the fragility of the specimens and the need for careful handling, but also because tumour heterogeneity can result in a biopsy that does not represent the whole tumour [4, 53, 54].

A critical issue that remains to be solved is distinguishing between aggressive and low-risk UTUC preoperatively to serve as indications for KSS or radical surgery. Other prognostic factors such as MSI and tissue-based molecular markers have been studied [55], and MSI typing has been shown to aid detection of germline mutations and hereditary cancers [20]. The validation of molecular prognostic biomarkers in biopsy specimens would be invaluable in treatment decision-making. Thus far, most studies of tissue-based molecular markers have primarily used RNU specimens, although Bagroida et al. [56] found high concordance in genomic alterations between tumour biopsies and subsequent RNU specimens. None of the investigated markers, neither MSI nor tissue-based molecular markers, have yet fulfilled the criteria necessary to support their introduction in daily clinical decision-making. However, it is plausible that incorporating tissue-based markers in prognostic tools in the future can help identify patients who would benefit from KSS or intensified therapy and monitoring.

The literature concerning long-term results of different treatment modalities for UTUC is vast and confusing, and most studies have been retrospective. Today, RNU is the treatment of choice in all cases of high-risk organ-confined disease, but RNU entails a significant risk of CKD and haemodialysis, the latter of which is associated with increased morbidity and mortality [10]. In low-grade non-invasive UTUC, endoscopic treatment seems to be not only equivalent to RNU with regard to oncological outcome, but also saves renal function. Hurel et al. [57] found that CSS was similar in patients after KSS or RNU, but that OS was better after KSS, possibly due to reduced renal function in the RNU group. Lee and co-workers [58] compared renal function in 319 patients treated with radical nephrectomy and 297 patients who underwent RNU, and found a greater than threefold higher risk of doubling of Cr or dialysis in the RNU patients.

Endoscopic treatment has high local recurrence rates, and, in some cases, progression of the disease leads to a later RNU. Nevertheless, it may take years for progression to occur, and endoscopic treatment can save patients from long-term suffering with CKD [6, 30]. Increased rates of bladder recurrence after diagnostic URS have also been reported, and a single dose of intravesical mitomycin postoperatively has been discussed. Notably, a systematic review and meta-analysis conducted by the EAU guidelines panel [59] showed that there are a number of predictors of intravesical recurrence, which are patient specific, tumour specific, and treatment specific, but not necessarily linked to preoperative diagnostic URS. Hence, post-URS mitomycin instillation is still a matter of debate.

The overall goal of risk stratification is to select the best option for long-term health. That process entails two tasks: the first is to make an accurate diagnosis, and the second is to determine the best personalized treatment that will lead to a tumour-free status. If KSS is chosen, close follow-up with repeated URS and CTU is necessary, possibly for the rest of the patients’ lives. Endoscopic management requires ‘buy-in’, in other words commitment to long-term surveillance to detect and treat any recurrence. On the other hand, radical treatment can lead to CKD. Clearly, the patients must be well informed about the advantages and adverse effects of both radical treatment and KSS.

## Conclusion

Risk stratification in UTUC is feasible and should be done in all cases of diagnosed UTUC. The prognostic markers used in clinical practice are based primarily on the results of retrospective and small studies. To date, the strongest preoperative prognostic markers are tumour grade and indirect signs of invasiveness and the best way to ascertain these preoperatively is URS with in situ samples for cytology and histopathology. Investigations of new prognostic markers are ongoing, and, hopefully, in the future, diagnostic genomic profiling will aid treatment decisions and personalized therapeutic approach. KSS seems to have advantages over RNU for the treatment of low-grade non-invasive tumours but there is a need for prospective long-term follow-up studies to confirm this. Moreover, KSS requires multiple follow-up procedures, which many patients prefer to avoid and, therefore, we need to develop refined models for risk stratification and for follow-up.
